# Supplementary figures and images for: Differing physiological and behavioral responses to anthropogenic factors between resident and non-resident African elephants at Mpala Ranch, Laikipia County, Kenya
Source: PeerJ. 2020 Sep 28;8:e10010. doi: 10.7717/peerj.10010 (PMC7528812; doi:10.7717/peerj.10010)

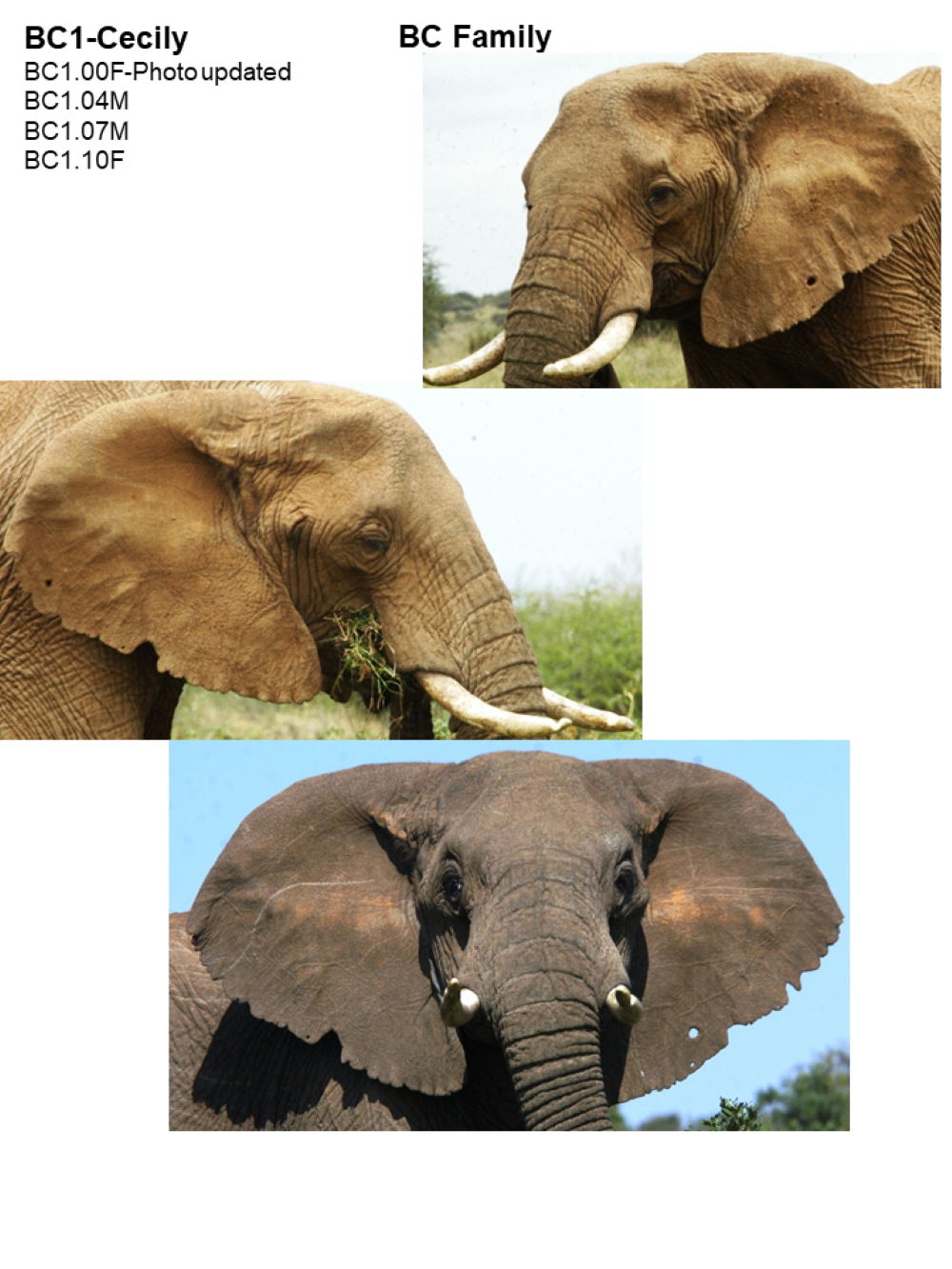

Supplement: Supplemental Information 3 — A sample of the catalog recognition file for BC family group for monitoring elephants showing the matriarch of the BC family (BC1) with four calves (BC1.00, BC1.04, BC1.07, BC1.10). Sex of the calves are represented as female (F) or male (M). Inverted ‘U and V’ shape cut on both sides of the ears as well as the holes were used to distinguish it from other individuals. [file peerj-08-10010-s003.png]

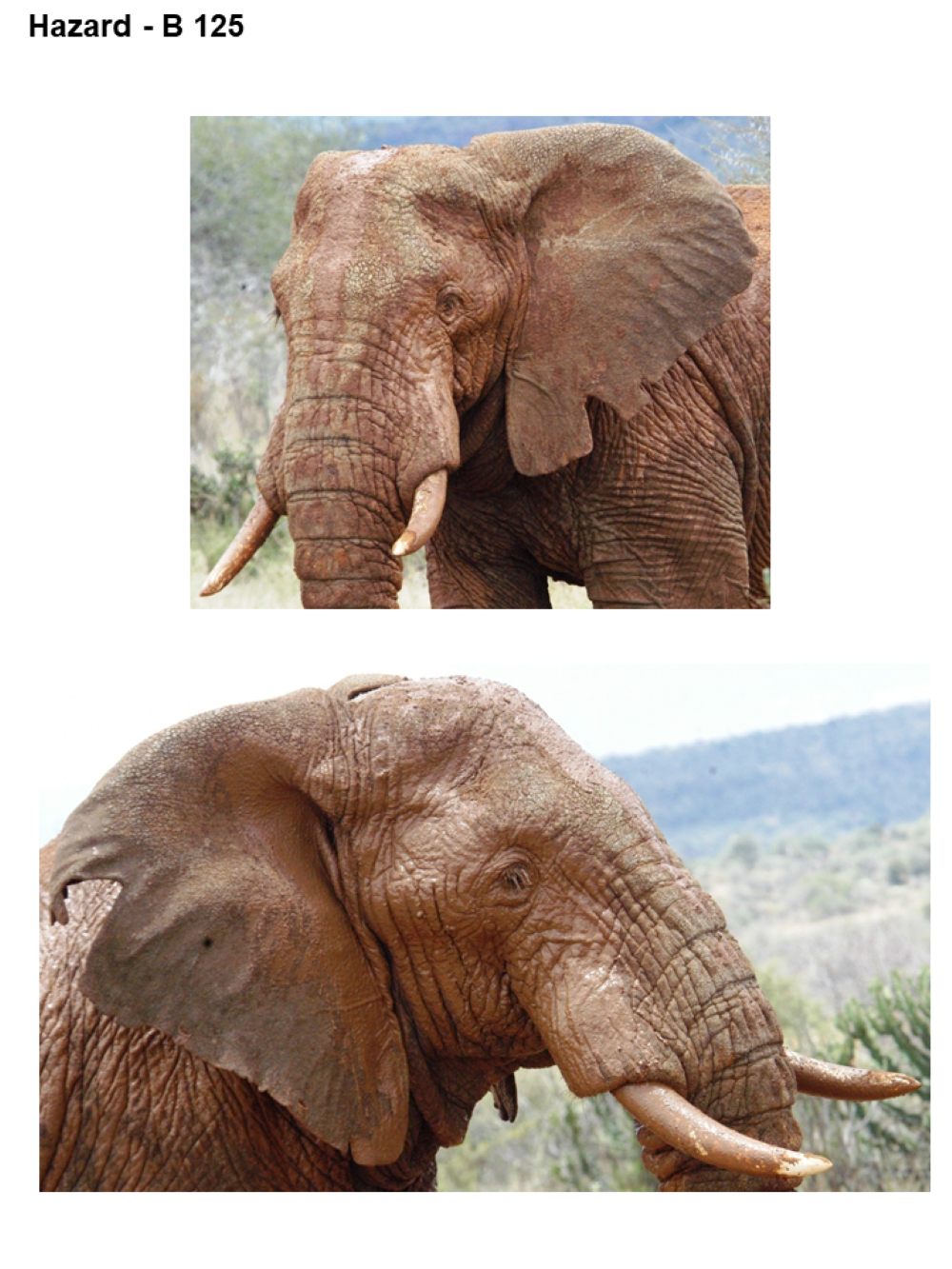

Supplement: Supplemental Information 4 — A sample of an adult bull in the catalog recognition file for monitoring elephants at Mpala ranch. Hazard B – 125 refers to 125th adult bull and named Hazard to be recorded onto the Mpala elephant database. Deep ‘V’ cuts on both sides of the ears were used to distinguish it from other bulls. [file peerj-08-10010-s004.png]

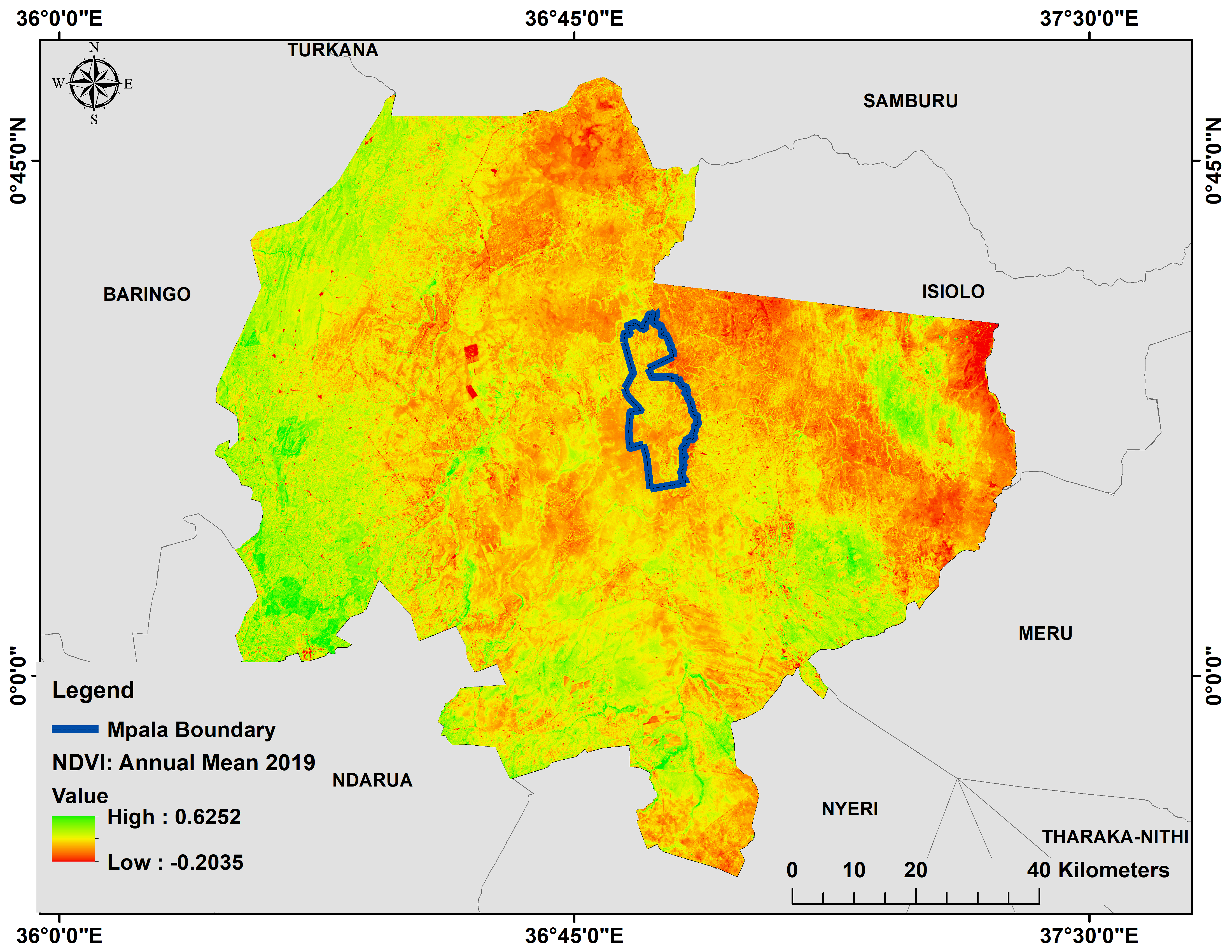

Supplement: Supplemental Information 5 — The graph shows a comparison of an annual mean 2019 NDVI base layer for Mpala Ranch (in blue) preferred by the resident elephants and within Laikipia where non-resident traverse. [file peerj-08-10010-s005.png]

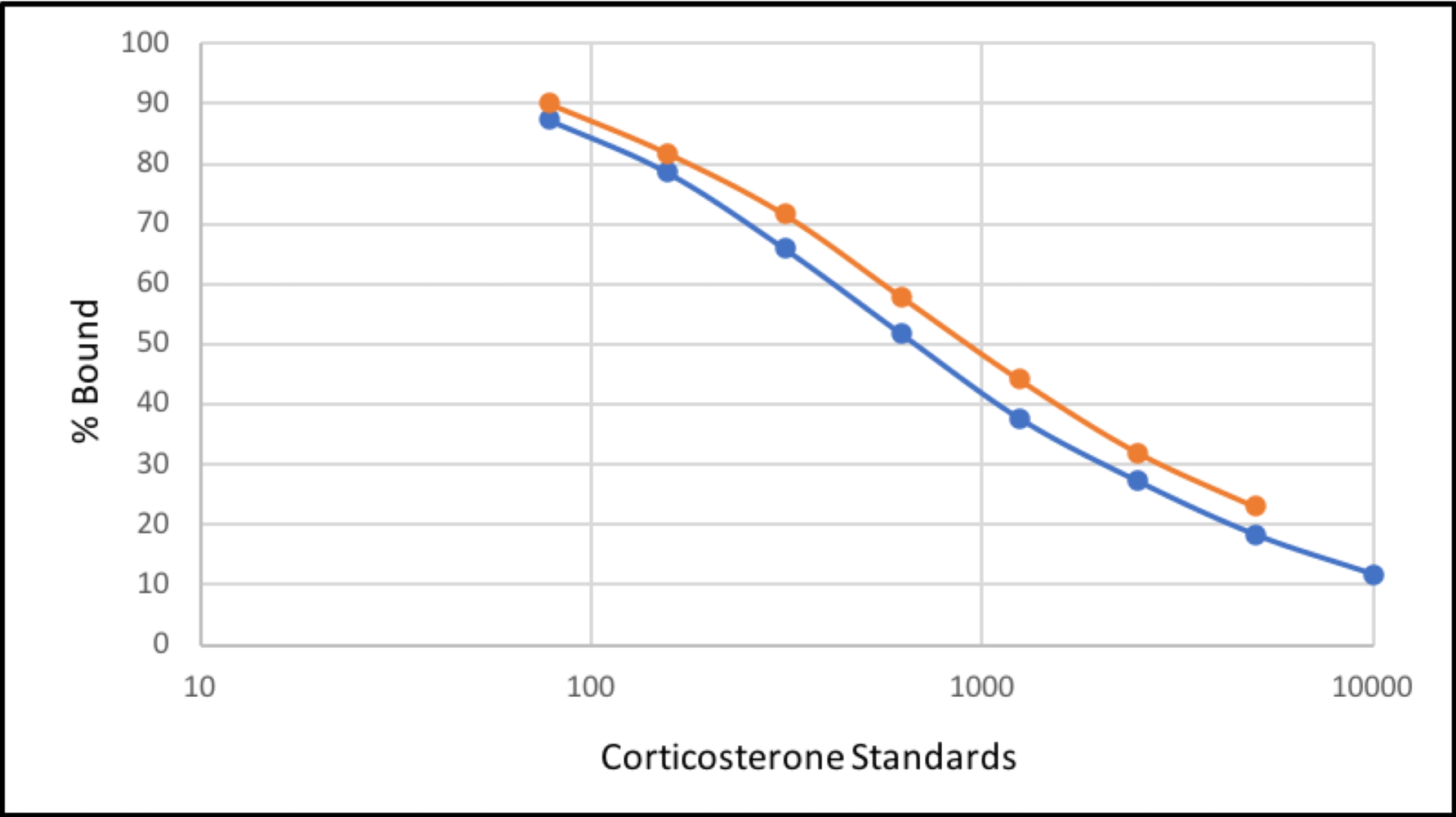

Supplement: Supplemental Information 6 — A graph showing parallelism between serial extract dilutions and the standard curve. [file peerj-08-10010-s006.png]

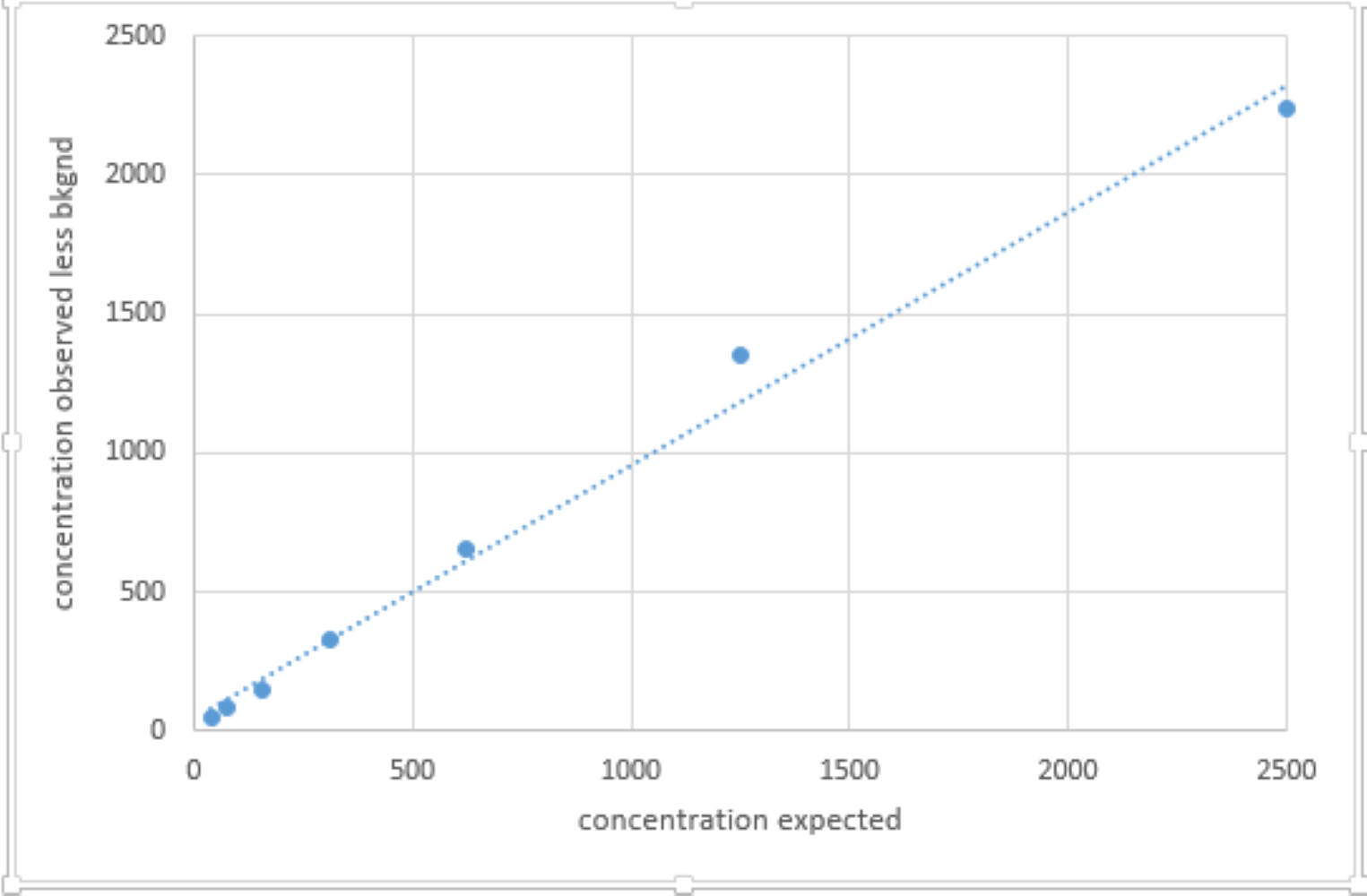

Supplement: Supplemental Information 7 — Significant recovery of unlabeled corticosterone standard added to a low concentration sample. [file peerj-08-10010-s007.png]

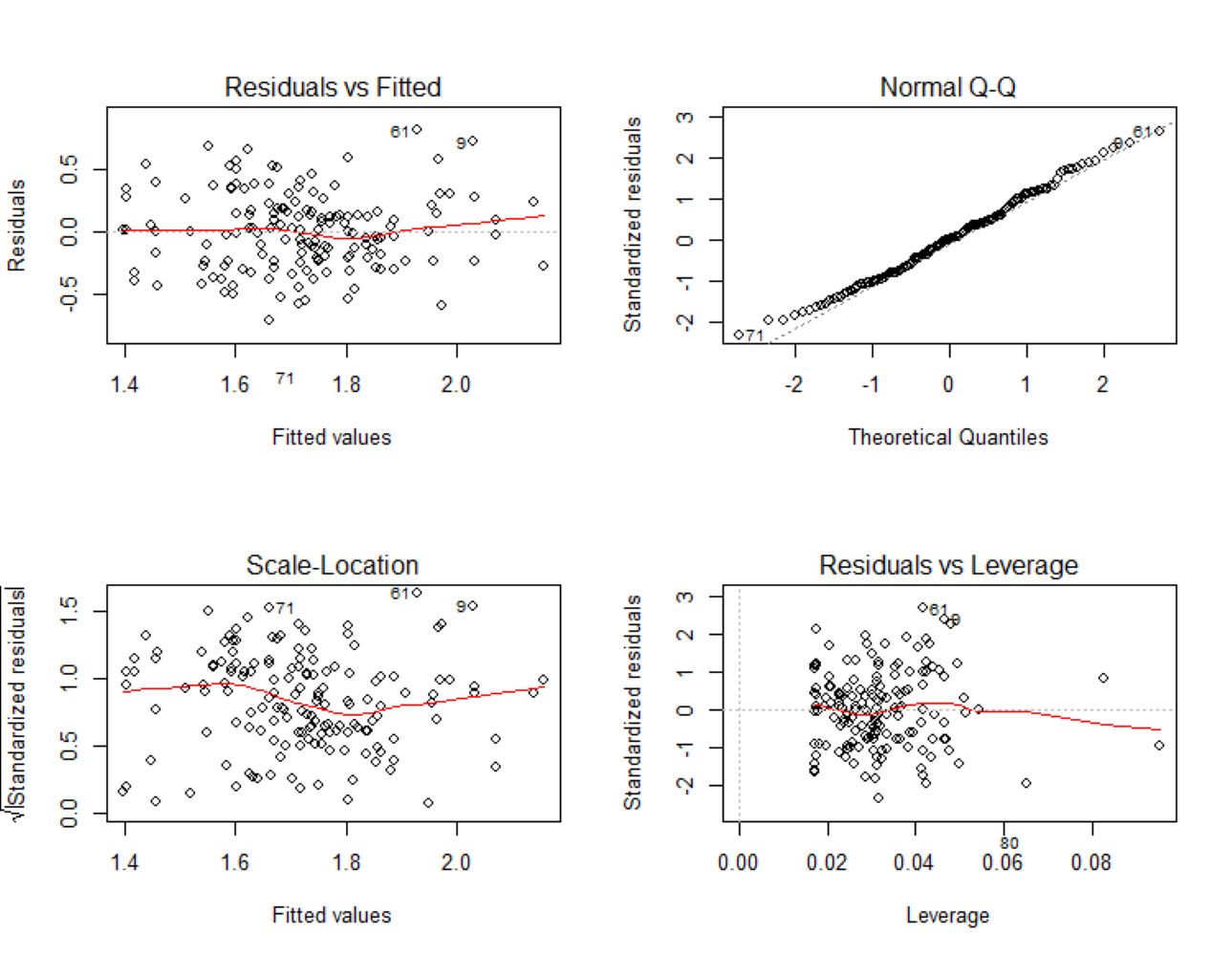

Supplement: Supplemental Information 8 — A plot of the model showing linearity, normality of the residual, homogeneity and independent sample points. [file peerj-08-10010-s008.png]

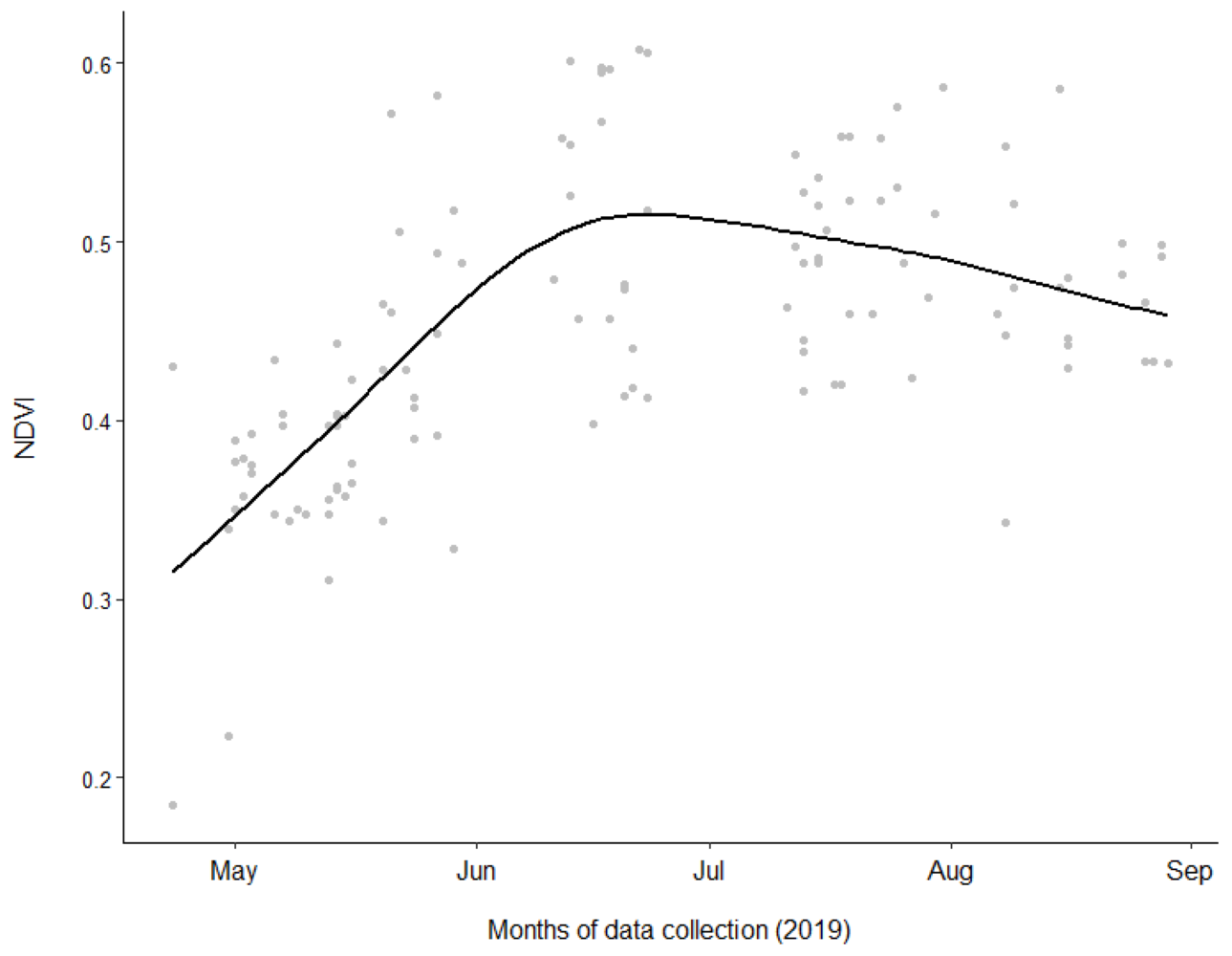

Supplement: Supplemental Information 9 — A non-linear curve showing the fluctuation in NDVI values of fecal data points during the months of data collection. [file peerj-08-10010-s009.png]

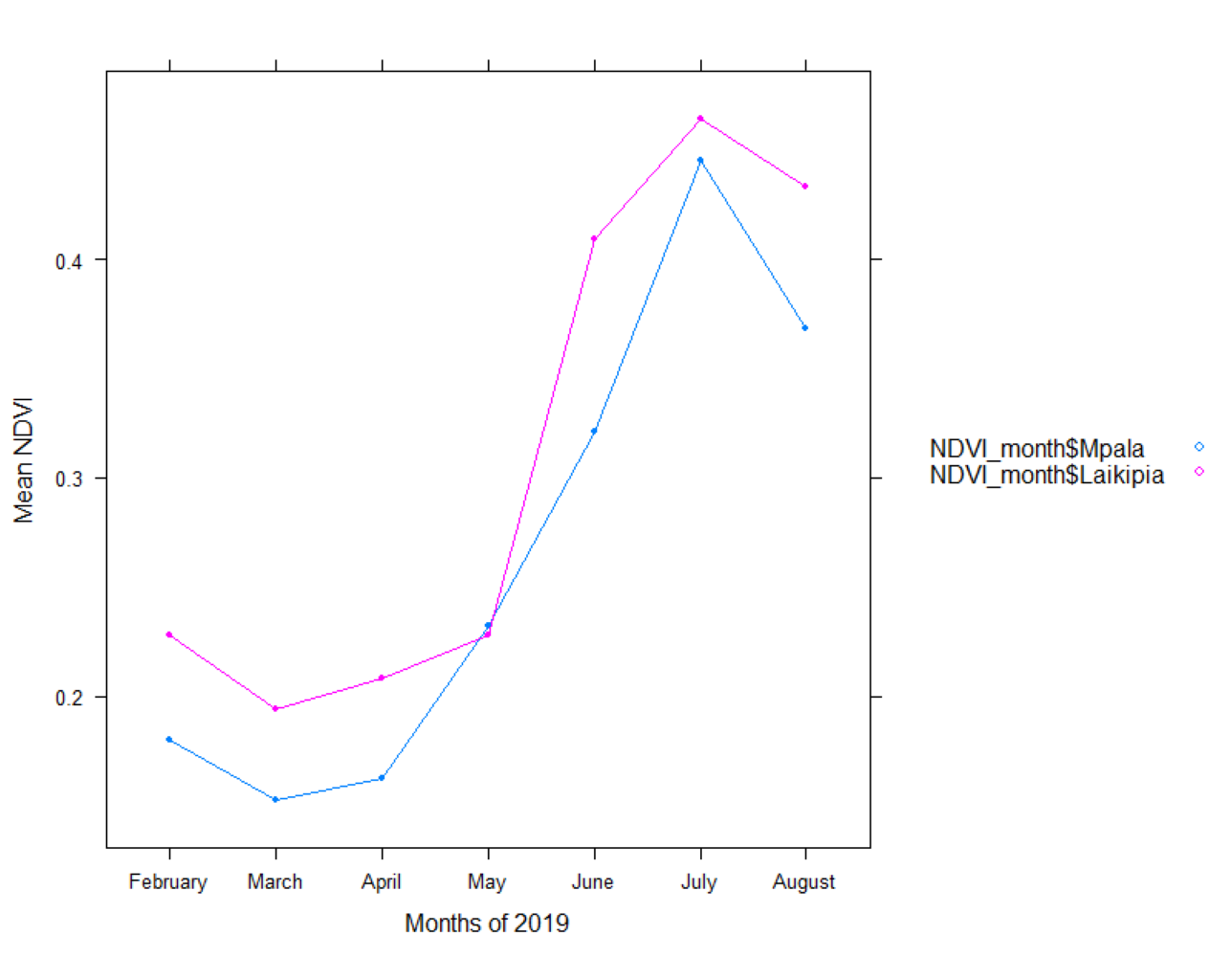

Supplement: Supplemental Information 10 [file peerj-08-10010-s010.png]
